# Understanding leprosy reactions and the impact on the lives of people affected: An exploration in two leprosy endemic countries

**DOI:** 10.1371/journal.pntd.0010476

**Published:** 2022-06-13

**Authors:** Annisa Ika Putri, Kevin de Sabbata, Regitta I. Agusni, Medhi Denisa Alinda, Joydeepa Darlong, Barbara de Barros, Stephen L. Walker, Marjolein B. M. Zweekhorst, Ruth M. H. Peters

**Affiliations:** 1 Athena Institute, Faculty of Science, VU University Amsterdam, Amsterdam, Netherlands; 2 Department of dermatology, Dr. Soetomo General Teaching Hospital, Surabaya, Indonesia; 3 School of Law, Keele University, Keele, Newcastle, United Kingdom; 4 The Leprosy Mission Trust India, New Delhi, India; 5 Department of Clinical Research, Faculty of Infectious and Tropical Diseases, London School of Hygiene and Tropical Medicine, London, United Kingdom; Emory University School of Medicine, UNITED STATES

## Abstract

**Background:**

Leprosy reactions, Type-1 and erythema nodosum leprosum, are immune-mediated complications of leprosy, which play a significant role in the morbidity associated with the disease. A considerable amount of literature has been published on the impact of leprosy in general but few studies focus specifically on leprosy reactions. This study aimed to investigate the impact of leprosy reactions on physical, psychological, and social aspects of the lives of people affected by analysing their life experiences and perspectives about leprosy reactions.

**Methods/Principal findings:**

This qualitative study involved people affected by leprosy reactions and their family members in two leprosy endemic countries. The data were collected through 66 interviews and 9 focus group discussions (4–6 participants each) in Surabaya, Indonesia, and Purulia, India. Content analysis and conversational analysis were performed. This study found that both types of leprosy reactions were perceived as an unpredictable and painful condition. Leprosy reactions restricted physical activities of the participants, such as going to bathroom, sleeping, eating, and cooking. In the interviews, the respondents expressed a range of emotions and feelings including confusion, sadness, anxiety, and anger. Some recounted that they felt stigmatized and lost opportunities to socialise and earn money. Differences between the two settings were identified. The majority of Indonesian participants preferred to stay at home, and some concealed the diagnosis of leprosy, while most of the Indian respondents continued working up to the time of hospitalization.

**Conclusion:**

Leprosy reactions are a distressing complication of leprosy and adversely affect the lives of those affected. Individuals reported physical discomfort, distress, anxiety, stigma, and financial hardship and these negative impacts in the physical, psychological, and social spheres reinforced each other. These findings provide important information about a need for early detection and sustained commitment to follow-up care for people with a history of leprosy reactions. More research on new drugs for reactional episodes, tools to measure knowledge, attitude, and practice, and costing study on leprosy reactions treatment are needed. We recommend the development and testing of holistic strategies to improve the management of leprosy reactions.

## Author summary

Leprosy reactions are immune-mediated complications of leprosy. There are two types of reaction. To provide a deep understanding about experiences and perceptions of people with both types of reactions, we interviewed the individuals affected and conducted group discussions with their family members in two leprosy endemic countries: Indonesia and India. The results of this study show that both types of reactions significantly affect the participants’ lives. People with leprosy reactions in both countries experienced persistent pain which hampers their daily activities. A majority of participants reported that this makes them feel more often sad, that they isolate themselves, rely upon their family members and have less income. There is a need for more support for people with leprosy reactions and we recommend testing of different interventions including (peer) counselling.

## Introduction

Leprosy is a neglected tropical disease caused by *Mycobacterium leprae* and *Mycobacterium lepromatosis*. It mainly affects the skin, peripheral nerves, upper respiratory tract mucosa and eyes [1]. Globally, there were 202,185 new cases of leprosy reported to WHO in 2019 [2]. The country with the highest number of reported cases was India (114,451), followed by Brazil (27,863) and Indonesia (17,439) [2]. Ridley and Jopling classified leprosy into five groups based on its clinical and histological criteria ranging from tuberculoid leprosy (TT), which is characterized by few flat lesions and some nerve involvement, to lepromatous leprosy (LL), which is identified by more lesions and more nerve involvement [3,4]. The WHO categorise leprosy as paucibacillary with, five or fewer skin lesions and multibacillary with six or more skin lesions [3,5].

Leprosy reactions are immune-mediated complications of leprosy which play a significant role in the morbidity of the disease [6–8]. Leprosy reactions are classified into two types [9]. Type-1 reactions (T1R) are characterised by the development of acute inflammation in skin lesions and/or nerves [9,10]. Erythema nodosum leprosum (ENL) or Type-2 reactions are characterised by fever with crops of painful, erythematous, cutaneous nodules with individuals frequently experiencing multiple episodes [9]. T1R and ENL may cause severe and irreversible nerve damage, thereby contributing to disability and should be treated promptly [1,7,11].

T1R and ENL may occur before, during or after completion of antimicrobial multidrug therapy (MDT). Studies have reported a variety of proportions of persons affected by leprosy who develop leprosy reactions. The type of leprosy is an important risk factor with T1R affecting between 20–40% of individuals with multibacillary (MB) leprosy [12–17]. T1R frequently occur after starting MDT and its duration depends on the clinical form [18,19]. ENL reactions occur in people with borderline lepromatous (BL) leprosy and LL. Approximately 10% of people with BL leprosy and up to 50% of those with LL are estimated to develop ENL [20,21]. ENL can be distinguished in three types which are single acute ENL, multiple acute ENL (repeated discrete episodes), and chronic ENL (continuous episodes) [21]. ENL is commonly seen during the first year of MDT [7,21–23]. Both T1R and ENL require corticosteroids or other immunomodulatory drugs, in addition to MDT for the infection [8,24]. However, there is no consensus about dose and duration of treatment with corticosteroids [18]. MDT and additional clofazimine (for severe ENL) is provided free by the World Health Organization, but other treatments must be paid for by people with leprosy and leprosy reactions [25–27]. In a leprosy endemic country, Brazil, thalidomide is chosen as a therapeutic regimen for ENL which complies with the country legislation [28]. In addition to the pain and neurological (motor and sensory) impairments, leprosy reactions are problematic because they are perceived as an indication of ineffective antimicrobial treatment with MDT [29]. This is worrisome during MDT, but also after completion of MDT, as a study in Brazil showed that persons affected can lose their faith in the fact that leprosy can be cured and that leprosy reactions can be controlled [29].

Most studies on leprosy reactions focus on pathophysiology, clinical and epidemiological aspects [8,20,30,31] but four have assessed the associated health-related quality of life [32–35]. The specific factors which influence quality of life, how people face adversity, and to what extent social and personal factors, as opposed to medical ones, play a role in them have not been explored in these studies. To our knowledge, there is a lack of a thorough, in-depth analysis of the impact of leprosy reactions on the lives of affected individuals which considers physical, psychological and social dynamics. Moreover, there is only one study with participants from Brazil, that has given the necessary centrality to the voice of people with leprosy reactions [29]. Research is needed for a deeper understanding of the life experiences and impact on quality of life of leprosy reactions. The objective of this study was to investigate the impact of leprosy reactions on physical, psychological, social aspects of the lives of people affected by analysing their life experiences and perspectives about their condition in India and Indonesia.

## Methods

### Ethics statement

Ethical approval for this study was granted by the ethical review committees of Dr. Soetomo General Hospital (070/91/301.4.2/Litb/II/2019), the Leprosy Mission Trust India (5/vii/C-37), and London School of Hygiene and Tropical Medicine (17007). Participants gave written informed consent before the data collection began. Formal consent was obtained from the parent/guardian (including whether it was verbal or written) for those participants under 18 years old.

#### Study Design

A qualitative design was selected for this study. To provide thick descriptions and a rich context on findings, this research incorporated comparative methods in which we compared the experience reported by participants from each country and then categorized it based on the study location and its socio-cultural factors [36]. The study settings were Dr. Soetomo General Hospital, Surabaya, West Java, Indonesia and The Leprosy Mission Home and Hospital, Purulia, West Bengal, India. Participants were adults (over 16 years) who were experiencing leprosy reactions or had experienced them within the previous 12 months and their family members (e.g., spouse, parent, sibling). Purposive sampling was used considering characteristics such as gender, age, and type of leprosy reaction (S1 Appendix). The data collection activities were stopped after data saturation was achieved.

#### Data Collection

Data were collected using interviews and focus group discussions (FGDs). Engel’s biopsychosocial model [37] was used as framework to develop the interview guide (S2 Appendix). The framework conceptualizes the interrelation between a disease and biological, psychological, and sociological dimensions of an individual’s life [37]. Six people affected by leprosy and leprosy reactions provided input on the topics that should be explored and how to formulate the questions for the interview guides during a workshop in Indonesia. Two people with leprosy reactions provided input about topics for interview guides during personal conversations in India. During these workshops and conversations, needs and interests, appropriateness of the topics and the interview questions were discussed. The final interview guide explored the general experience of living with leprosy reactions (e.g., when did they notice the symptoms and what did they do) and progressed toward more in-depth questions on the biopsychosocial impact of leprosy reactions (e.g., activities they are able or unable to do, feelings, stigma). In the FGDs, family members discussed their experiences, needs, worries related to taking care of relatives with leprosy reactions, family finances and their relationship with the affected individuals. The guides for Indonesia and India differed slightly. In India, pictures of drugs and a body map from National Leprosy Program were used to facilitate discussion.

Data were collected from January to June 2019 in Indonesia and from July to September 2019 in India. In Indonesia, we collected the data in a general hospital and urban setting, whereas in India it was conducted in a leprosy-specialized hospital and rural setting. Interviews and FGDs were held in private either at the hospital or participants’ homes. The interviews and FGDs, lasted on average an hour and were conducted by the first author (AIP) and two research assistants in Indonesian, Hindi, or Bengali. The Indonesian research assistant had personal experience of leprosy reactions. The Indian research assistant comes from a leprosy-affected family. Follow-up questions were asked to participants through phone or in-person interviews when necessary.

All interviews and FGDs were audio-recorded, transcribed, and then translated into English. Pseudonyms have been used in this manuscript for participants who are quoted. Data were managed and analysed using NVivo 12. Content analysis and conversational analysis were performed. The transcripts were coded and categorised based on the biopsychosocial model [37]. Expressions used in the interviews were also analysed and classified based on their meaning. The analysis was done iteratively in consultation with the research assistants. After the data from Indonesia were analysed, we presented the preliminary findings to a selection of the interview and FGD participants in two workshops in Indonesia. The people affected by leprosy reactions, family members, and healthcare professionals sat together, reflected on the results, and confirmed or clarified the findings. After the data from India were analysed, we validated the findings via phone interviews with 12 participants affected by leprosy reactions because the workshops were cancelled due to the COVID-19 pandemic.

## Results

### Characteristics of study participants

Sixty-six people affected by leprosy reactions were interviewed. Four Indonesian participants were related; they were two pairs of siblings. Most participants were men and married. Indian participants were slightly older than Indonesian participants. In Indonesia, most of the participants lived with their nuclear family, whereas in India half of the participants stayed with their extended family. Compared to Indonesian participants, there were more Indian participants who still worked while receiving treatment for leprosy reactions. All Indonesian participants were Muslim with diverse educational background. In contrast, the majority of Indian participants were Hindu and had not completed primary school. Table 1 provides more detailed demographic information of participants affected by leprosy reactions. The data saturation happened sooner for participants with T1R, than those with ENL which affects more organ systems and exhibits greater chronicity which makes it a more complex condition.

**Table 1:** Demographic of participants affected by leprosy reactions in Indonesia and India.

Variables	Indonesia (n)	India (n)	Total
**Gender**			
Man	26	23	49
Woman	7	10	17
**Age group**			
16–25 years old	10	9	19
26–35 years old	12	9	21
36–45 years old	5	10	15
46 years old and above	6	5	11
**Current occupation**			
Unemployed/Retired	11	-	11
Housewife	5	6	11
Civil servants	3	-	3
Manual labour (e.g., farmer, construction worker)	6	20	26
Others (e.g., Private worker, merchant, self-employed, student)	8	7	15
**Marriage status**			
Married	22	28	50
Not married	11	5	16
**Highest completed level of education**			
Not completed primary school	-	14	14
Primary school	5	8	13
Secondary School	23	10	33
University	5	1	6
**Personal income**			
> 60 USD/month	19	24	43
60–150 USD/month	5	6	11
< 150 USD/month	9	3	12
**Religion**			
Muslim	33	2	35
Hinduism	-	31	31
**Currently living with**			
Nuclear family	22	17	39
Extended Family	9	16	25
Friends	1	-	1
Alone	1	-	1
**Delay of treatment from the first notified symptom leprosy**			
<6 months	15	17	32
6–12 months	2	4	6
>1 year	16	12	28
**Who knows about the diagnosis of leprosy**			
Nobody	0	1	1
Very few (1–3 people)	11	11	22
People in the household	14	9	23
Some extended family members, friends, and neighbours, but not all	7	4	11
Everybody	1	8	9

Fourteen participants experienced T1R and 52 experienced ENL. For the majority (n = 42) the first episode started during the MDT course. The reactions commonly emerged on the visible body parts, such as hands, legs, or face. Thirty-nine percent of the Indonesian participants had experienced 2–4 episodes of leprosy reactions, whereas 42 percent of the Indian participants had experienced >4 episodes of leprosy reactions (Table 2).

**Table 2:** Clinical Features of leprosy reactions of people affected in Indonesia and India.

Variable	Indonesia (n)	India (n)	Total
**Type of leprosy reactions**			
Type-1 reactions (T1R)	7	7	14
Type-2 reactions (ENL)	26	26	52
**When the first leprosy reactions occur**			
Before MDT started	7	10	17
During MDT	21	21	42
After MDT completion	5	2	7
**Number of leprosy reaction episodes**			
1 episode	8	10	18
2–4 episodes	13	9	22
>4 episodes	12	14	26
**Visibility of leprosy reactions**			
Visible on face, hands, arms, or feet	28	31	59
Invisible (located on covered parts of the body, e.g., stomach or chest)	6	2	8

In the FGDs, 16 Indonesian and 22 Indian family members participated representing 15 Indonesian and 17 Indian families, respectively. In Indonesia, the FGD participants were a parent (38%), spouse (31%), sibling (19%) or adult child (13%) of the participants with leprosy reactions. FGD participants in India were spouses (41%), parents (36%), adult child (18%), or sibling (5%) of the participants. In Indonesia, 12 (38%) and in India 5 (16%) FGD participants were women.

### Descriptions of illness

In both countries, the participants with leprosy reactions used lay words to illustrate how their body felt when leprosy reactions occurred. Common terms used in Indonesia are “*gringgingan*” [tingling sensation], “*crecep”* [burning sensation], “*kenyong kenyong”* [swollen] and *“cekot-cekot”* [sore]. Meanwhile, the participants in West Bengal, India, describe the leprosy reactions as “*Jinjin”* [tingling sensation]. The ideophones from both countries refer to uncomfortable feelings experienced during leprosy reactions. The participants mentioned pain, fever and cutaneous swelling and some individuals in Indonesia reported nausea and vomiting. The range of symptoms experienced was confirmed during FGDs with family members and validation activities. Participants indicated confusion and anxiety associated with the emergence of leprosy reactions. Participants were able to distinguish between leprosy reactions and non-reactional leprosy due to the inflammatory symptoms described as swelling and burning sensation. The tingling sensation and pain motivated participants to either seek medical advice or to try to reduce the discomfort by using alternative treatments.

### Timing of onset

Seventeen of sixty-six (25.75%) participants (seven Indonesians and ten Indians) developed leprosy reactions before leprosy was diagnosed and MDT started, 42 during MDT and 7 after MDT completion (Table 2). In two cases, reactions occurred long after they completed treatment; an Indonesian participant developed reactions more than 10 years and an Indian participant more than 14 years after completing MDT. The delay in onset of leprosy reactions is difficult to reconcile with the concept of cure of the disease.

*“I have no idea whether I have been fully recovered or not*… *It has been ten years [since the first leprosy symptoms]*. *I started to have the reactions since 2017 after completing MDT*. *They are getting severer”*. (Mustofa, ENL, Man, Teacher, 35 years old, Indonesia)

### Perceptions of aetiology and risk factors of leprosy reactions

Indonesian and Indian participants in interviews and FGDs made a connection between strenuous physical activity (e.g., cultivating land, construction work but also sports like running) (n = 6), fatigue (n = 11) and stress (in particular worrying) (n = 6) and the onset of reaction symptoms in the interviews and the validation activities. Leprosy reactions were attributed to cold temperature by two Indonesians and junk food consumption by another. Indian participants associated the illness with insect bites (n = 1), bathing in an unhygienic pond (n = 1) or giving birth (n = 1) and two participants linked leprosy reactions with alcoholism.

“*My whole body used to have burning sensation*. *My neighbours said that I had hathura, the local belief on sickness after giving birth*.” *(Gina, ENL, Woman, Housewife, 25 years old, India)*

“*If I carried a heavy load in the morning then the pain would start in the night*. *Then, the fever and tingling sensation will also come.” (Daksh, ENL, Man, Farmer, 21 years old, India)*

“*When I was exhausted after doing exercise like running, the nodules appeared*.” *(Arfan, ENL, Man, Civil Servant, 32 years old, Indonesia)*

“*The doctor said this is a spoiled/lazy [“ngalem”] person disease*. *It means that the pain relapsed if you were exhausted or thinking too much, but it would not appear if you were happy.” (Arfan, ENL, Man, Civil Servant, 32 years old, Indonesia)*

### Diagnosis of leprosy reactions

Seven Indonesian and ten Indian participants who developed leprosy reactions prior to receiving the diagnosis of leprosy often sought medical advice for the reactions and then learned they had leprosy complicated by leprosy reactions. An Indonesian participant assumed, in the interview, that delay in diagnosis of leprosy contributed to leprosy reactions. Delay in diagnosis of leprosy was mentioned often in the interviews. Twenty-eight participants (42%) said there was a delay of more than a year (see Table 1). Eleven Indonesian and fifteen Indian participants reported that the medical doctors at their local health care facilities initially made a diagnosis such as dermatitis or dermatophytosis or allergy. Leprosy reactions which developed following the diagnosis of leprosy and during MDT were diagnosed quickly and was used as a confirmation of the diagnosis of leprosy at health facilities.

“*At first, I thought it was an allergy due to I like eating junk foods*. *Then, I had nodules and pus on my legs. A doctor in Kediri suggested me to visit leprosy hospital and I got MDT and a prescription of methylprednisolone [for treating the leprosy and leprosy reactions]”. (Bambang, ENL, Man, Unemployed, 27 years old, Indonesia]*

Participants who developed leprosy reactions after completion of MDT reported delays in the diagnosis of the reactions. Health care professionals did not always inform the patients about the possibility of developing leprosy reactions, hence some participants delayed seeking care as they thought the condition was harmless and not related to leprosy. They only sought help when the symptoms worsened. Misdiagnosis at this stage by health professionals also occurred.

### Impact on physical sphere

Direct and indirect effects of leprosy reactions on the physical body were identified. Bodily functions were directly affected by leprosy reactions and indirectly affected by the treatment of leprosy reactions. Problems with bodily functions had a negative effect on daily activities and participation and were reported by participants and family members.

The neuritic pain and burning sensations associated with leprosy reactions created difficulty with movement and muscle strength. Difficulties with moving arms, lifting legs, bending knees, or holding things were reported. The participants in both countries, confirmed by family members during the FGDs, said that fatigue contributed to problems with bodily functions. Most of the participants expressed the idea that symptoms worsened when they felt fatigued after performing strenuous activities. In some cases, bodily functions were severely affected.

“*It was excruciating like ants bit my legs. I cried*. *I couldn’t get a good sleep. The pain was persistent” (Sinta, T1R, Woman, Housewife, 50 years old, Indonesia).*

The problems with bodily functions created problems with daily activities like self-care, eating and household tasks but once the inflammatory symptoms settled levels of activity normalised.

*“It was all swollen on my arms and legs*. *I could not even comb my hair”*. (Lina, T1R, Woman, Student, 19 years old, Indonesia)

*“When the pain relieved*, *I did all kind of work like cultivating the land or picking up water from the well*. *Yet*, *it was not possible to do these activities when the nodules come out along with the pain and fever*. *Due to this*, *I was not able to eat*, *sit and bend my knee*.” (Chaarun, Woman, ENL, Farmer, 35 years old, India)

The type of leprosy reaction, the severity of the reaction, finance, and support from household members all influenced the extent of the impact within the physical sphere. Participants in both settings described the pain associated with ENL as more severe than that associated with T1R. The majority of participants with ENL in both countries were incapacitated (bed bound) when the ENL was at its most severe, whereas participants affected by T1R did not experience this level of incapacity due to pain.

Indirect effects of leprosy reactions on bodily functions were caused by medical therapy. The drugs to treat leprosy reactions caused adverse effects that influenced bodily functions according to participants. The most important adverse effects reported by participants were anaemia and skin discoloration. Half of the participants in Indonesia and India said in the interviews that they experienced anaemia during MDT and the treatment of leprosy reactions, and many of the individuals were taking iron tablets in addition to the treatment for leprosy reactions. Other adverse effects reported were increased blood glucose levels, abdominal pain and palpitations.

### Impact on psychological sphere

Participants from both countries described the upset and surprise they experienced with the sudden changes that appeared on their hands, face, or legs. Some of participants became tearful when they were asked about the experience of having leprosy reactions during the interview. Participants expressed a wide range of negative emotions and feelings including sadness, fear, despair, anxiousness, anger, and confusion. The family members during the FGDs confirmed this initial shock and reported range of feelings. The participants said that they were optimistic only when the treatment reduced the severity of reactional skin lesions and the burning sensations. Participants with ENL reported more challenges and concerns regarding their illness due to the direct and indirect physical effect of leprosy reactions, and there was a bigger impact on their psychological well-being than on those participants with T1R who experienced less physical discomfort and no incapacitating pain. The range of feelings reported were elicited by several factors; the six main ones were appearance of leprosy reactions, limited activities, unpredictability of the reaction episodes, slow progress of treatment, fear of death and concerns about polypharmacy.

The appearance of leprosy reactions frightened those affected. The fear pushed participants to stigmatize themselves and conceal their symptoms. Most participants said that only their nuclear family knew about their leprosy reactions. In general, we saw that Indonesian participants feared the condition more than Indian participants. The majority of those who were frightened discussed this with their healthcare provider, who tried to give medical reassurance by saying that eventually the skin will be normal again. The continuous burning sensation which limits activities and restricts participation predisposed individuals to negative feelings.

“*I used to cry with pain, fever and my weakness*.” *(Baasim, ENL, Man, Hard Labor, 35 years old, India)*

The lack of predictability of the condition and its episodes were reported by twelve Indonesian and ten Indian participants. They described that the onset of reactions can happen in the morning, afternoon, or night. The participants postponed their work or school if the reactions happened during the day and had sleep deprivation if the reactions occurred at night. Five Indonesians and two Indians had new episodes of reactions years after they were discharged from outpatient care for their leprosy reactions. Two-thirds of Indonesian and Indian affected by multiple episodes of ENL expressed feelings of apathy.

“*Sometimes, I wanted to be angry, but what could I do*? *To whom should I be angry?*” *(Bakri, ENL, Man, Unemployed, 24 years old, Indonesia)*

Participants were anxious if the response to treatment was slow but if skin lesions and nerve pain improved, they felt optimistic. In India, the severity of ENL and prolonged requirement for treatment caused a participant to consider self-harm.

“*Sometimes, I wanted to take poison and finish my life*,” *(Raghu, ENL, Man, Farmer, 64 years old, India)*

From the interviews, we found that three Indonesian participants and five Indian participants expressed a sense of hopelessness and felt that the leprosy reactions affecting them were incurable. Due to the regular episodes of nerve pain, they started to doubt the effectiveness of the treatment.

“*I was frustrated of visiting several hospitals and consuming many kinds of medicines*. *Yet, these gave no improvement in my health.” (Madhavi, ENL, Woman, Housewife, 40 years old, India)*

“*I am discouraged because I cannot be recovered from the treatment. Could you imagine? I have been treated for more than three years*.” *(Bima, ENL, Man, Unemployed, 29 years old, Indonesia)*.

A few participants in both countries were worried that they might die because of leprosy reactions. An Indonesian and two Indian informants explained that fear of death was associated with severe skin lesions.

“*Sometimes, I feel that this is my end, and I am not going to live anymore*.” *(Madhavi, ENL, Woman, Housewife, 40 years old, India)*

Although Indian participants worried about loss of income and separation from their family, six were optimistic about their treatment in the specialist leprosy hospital because they experienced improvement and they encountered less challenges due to hospitalisation than Indonesian participants with severe leprosy reactions who were treated on an outpatient basis.

A lack of information, incomplete information, and inconsistent messages from health professionals about the prognosis of leprosy reactions generated anxiety and despair.

“*A doctor previously told that this [leprosy reactions] could be cured completely. But the other days, another doctor explained that full recovery is not possible*.” *(Lina, T1R, Woman, Student, 19 years old, Indonesia)*

The anxiety due to lack of information about their illness lead four Indonesian participants to seek information from the internet and five others consulted with health professionals through WhatsApp messaging or phone calls. Only one Indian participant used the internet to find information about leprosy reactions and the others relied on their doctors for information about their illness.

Participants in both countries also perceived that taking multiple medications might be harmful such as causing renal damage. The people affected by T1R or ENL reported being prescribed at least three types of drugs to treat their leprosy reactions. In addition to oral corticosteroids, analgesics, iron, and vitamin supplements were prescribed. High doses of oral corticosteroids would be divided. Participants said they applied emollients to dry or scaly skin. The concern about the number of drugs prescribed had a negative impact on psychological state of the participants in both countries.

### Impact on social sphere

Leprosy reactions affected the social sphere, either directly or through physical and psychological changes. Impact in the social sphere also affected the physical and psychological spheres. Three main themes emerged: relationship with the individual’s family, stigmatization and socio-economic situation.

The impact of leprosy reactions was felt by the families of the persons affected. Almost all people affected by leprosy reactions, at some point, were housebound and had no choice but to rely on their spouse, parents, or children. During the FGDs with family members and the validation activities with participants, we learned that the family members did not understand what leprosy reactions were and that they referred to reactions as leprosy. The common response of the family members during the FGDs was *“why are you not recovered yet*?*”* and some family members thought the persons affected used reactions as an excuse to not participate in daily activities. In one case, an Indonesian man with leprosy reactions was left by his wife and son due to this issue. Most nevertheless were willing to support their family member affected by leprosy reactions.

From the interviews and FGDs we learned that about one third of the persons affected in our study were dependent on family members with daily activities such as eating, washing, and using the lavatory when the reactions were severe. Family members in both countries also assisted the persons affected while visiting healthcare facilities, purchased medicines, and encouraged them to adhere to the treatment. Five Indonesian individuals used complementary therapies–such as Jamu, a traditional medicine prepared from herbs and may consist of turmeric and/or ginger–in addition to the allopathic treatment prescribed. The five participants reported that Jamu helped them to feel better. Complementary therapies were less commonly used by Indian participants. In some cases, family members also supported persons affected financially. In a few cases, family members did not support the person affected. Bima was asked by his wife, with whom he has a child, to move back to his parents because he could no longer provide financial support for the family.

Interview participants described a difference between leprosy-related stigma and stigma secondary to leprosy reactions and that stigmatization impacted on the physical and psychological sphere. In some cases, leprosy signs had not been noticeable, or the participants had been able to conceal them, but the leprosy reaction associated skin nodules were in most cases obvious to others. Self-stigma related to skin discoloration due to consumption of clofazimine for treating ENL was reported by five Indonesians and three Indians. Participants mentioned the recurrent feelings of inability to discharge their roles and responsibilities in their family and society. The visibility of symptoms and sense of inability to accomplish their work resulted in feelings of shame or being less worthy. In India, a woman with leprosy reactions recounted that her mother-in-law often cursed her because of the visible skin nodules and her incapacity to manage household chores because of her “*weak body”*. In Indonesia, multiple individuals with visible nodules said that many of their neighbours avoided them and even encouraged others to do the same. Participants were stigmatized when using public transport.

“*When you walk, people start to move away from you, that just hurtful… In public transport, sometimes people moved away from me*. *What else could I do? I had this disease, and it’s just not possible to take public transport by myself with no one around”*. *(Sinta, T1R, Woman, Housewife, 50 years old, Indonesia)*

The stigmatization appeared to be more severe in Indonesia where most participants said they were advised to conceal of the signs of reactions by healthcare providers to reduce stigmatization and its negative effects. Some participants wore long clothes and masks to conceal skin nodules and wounds. One third of participants felt it was advantageous that the signs of leprosy reaction were not associated with leprosy by lay people and that this reduced the negativity they experienced. Affected individuals used words such as *“dirty blood” [darah kotor]*, *“black magic” [guna-guna]*, *“allergy”*, *“diabetes”*, *“liver problem”* to disguise the name of the condition. Eighteen Indonesian participants considered this was an effective concealment strategy because leprosy reactions were rarely found in their neighbourhood. Several participants in Indonesia explained that some caring neighbours and friends attributed the symptoms to food allergy and suggested the avoidance of certain types of food such as chicken and eggs. One person told us *“I complied*, *and then I became anaemic”* (Tirta, ENL, Man, Unemployed, 66 years old, Indonesia). In participants’ perception, the disguise and concealment reduced social isolation and rejection by others. The strategy prevented more questions about leprosy and, attributing the problem to allergy or another skin disease, resulted in sympathy and support from their neighbours or colleagues. Stigma was also reported by Indian participants, but some of them had no qualms disclosing their leprosy diagnosis and experience of leprosy reactions because several neighbours had been similarly affected by leprosy. They reported good support from their family members and peers in seeking effective treatment.

The participants in Indonesia and India experienced loss of income and increased expenditure on medication and transportation which created a financial burden for them and their families. This was confirmed during the FGDs and the validation activities. Participants from both countries with income less than 60 USD per month explained that they ate less nutritious food, were late with taking medicine, and forced themselves to work when it felt better not to. This complex situation led to anxiety which, in their point of view, further triggered the leprosy reactions and negatively influence their bodily functions. The financial hardship experienced by the participants affected the physical and psychological sphere. In both countries, most participants lost opportunities to earn money and pursue education due to severe pain. Some male participants in Indonesia and India said that their wives started working because they were not capable of doing so. Others continued working despite the pain and discomfort because they had no alternative. Some participants had to borrow money from their extended family or neighbours.

“*Due to this [leprosy reactions]*, *I resigned my job.” (Rutva, ENL, Man, Self-employed, 38 years old, India)*

“*I had to work with pain. As I am alone to do all of these works*, *I had no choice. Honestly, I felt very bad and painful.” (Sajan, ENL, Man, Hard Laborer, 35 years old, India)*

“*I used to borrow money from my neighbour for the treatment*.” *(Baasim, ENL, Man, Hard Laborer, 35 years old, India)*

The treatment of leprosy reactions, especially ENL, lasted for several months or years and required frequent consultations with healthcare providers. Many of those affected by ENL were advised to take bed rest.

Participants were anxious about the direct and indirect expenses of the treatment. Indonesian individuals without health insurance or those who were treated at a non-charitable hospital in India had to pay for treatment. Travel costs were also incurred. The medical care required was often located in tertiary level or a leprosy-specialised hospital increasing travel costs and expenditure on food and beverages for individuals and any accompanying persons. A man affected by ENL from Indonesia and another from India said that treatment was occasionally delayed because of financial hardship.

Ten Indonesian and twenty-three Indian participants reported that leprosy reactions and their treatment impacted their financial independence and ability to fulfil their responsibility as a breadwinner in the household. These participants were unemployed and could barely afford nutritious food while the leprosy reactions were being treated.

## Discussion

This study shows that leprosy reactions have a significant impact on the lives of those affected in physical, psychological, and social domains. Importantly, the interconnections between the physical, psychological, and social spheres further reinforce the adverse effects of leprosy reactions. Pain, skin changes, sense of hopelessness, stigma, financial hardship related to leprosy reactions have an individual and collective effect on wellbeing which is dynamic and may vary with treatment and duration of the reaction. To our knowledge, this is the first study with a qualitative approach, that focusses specifically on the impact of leprosy reactions on the lives of people affected and its dynamics in Indonesia and India. That leprosy reactions have a negative impact was already clear from a series of quantitative and qualitative studies conducted in Bangladesh, Malaysia, India, and Brazil [27,29,32–35,38], but the impacts and dynamics behind it were often not explored in depth.

Most of the participants in this study lacked important information about leprosy reactions. In general, the participants were unaware of leprosy reactions and associated symptoms until they were diagnosed. Unsurprisingly, seeking care and obtaining a diagnosis and starting treatment was delayed. Further delay was sometimes experienced due to misdiagnosis by health professionals. Several studies have reported delays in diagnosing leprosy [8,39–47]. Some studies specifically looked at leprosy reactions [8,46–48]. Raffe et al in Nepal found that of the 75 people with leprosy reactions, thirty delayed seeking help and remained untreated for up to two years [8]. Leprosy reactions were initially misdiagnosed as arthritis, photosensitivity, “nerve disease” or skin diseases in 65% of those presenting to non-specialised service [8]. Individuals presenting with leprosy reactions may have the diagnosis of leprosy delayed compared to individuals without the reactions due to lack of recognition of the clinical features [49–52]. Participants in this study believed that the delays resulted in worsening of the condition and more negative impacts on the physical, psychological, and social spheres, which has also been reported in Brazilian individuals with leprosy [53]. Delayed diagnosis is associated with increased rates of Grade 2 disability and individuals presenting with Grade 1 or 2 disability are more likely to have experienced leprosy reactions [54].

Leprosy reactions appeared to have a significant effect on the psychological sphere of participants. They often experienced a mix of negative feelings–upset, confusion, anxiety, despair and anger–because of the appearance and unpredictability of the leprosy reactions and prolonged treatment. This finding confirms results of quantitative studies of quality of life in people with leprosy reactions in Brazil, Bangladesh, Malaysia, and India [32–35]. The studies found that people with leprosy reactions, ENL in particular, have the worst score on psychological domains compared to people with non-reactional leprosy [32–35].

Several studies in India and Indonesia have explored the association between stigma and leprosy [39,55–62]. Stigma and stigmatization stems from perceptions of marginality and deviance in which individuals with undesirable characteristics in a social group are devalued [63]. Leprosy-related stigma often caused by visible changes of the disease, cultural and religious beliefs, fear of transmission, stigmatization due to leprosy reactions occurred via two different mechanisms: visible changes (skin discoloration, ulcerated skin lesions, and nodules)- and the inability to perform roles and meet responsibilities within the family unit. The development of skin lesions, nodules, and wounds induced enacted stigma by the affected individual’s family, neighbours, and other social contacts in both countries, but also contributed to perceived and internalized stigma in the persons affected. This finding supports the hypothesis of Sermittirong [64] and Van Brakel et al [65] that external manifestations of leprosy are one of the causes and determinants of health-related stigma. In Nepal, people with visible impairments (including ulcers) were shown to experience significantly higher levels of perceived stigma than those with no such physical changes [66]. The dynamics between stigma and being unable to fulfil responsibilities within the family have been explored less in leprosy-related stigma research. Yang et al have shown that the inability to participate in the activities that ‘matter most’ in a cultural context threatens an actor’s personhood and can increase health-related stigma by blocking individuals from fulfilling what is most valued within a cultural context such as providing for the family [67,68]. In this study, the missing value or responsibility within the family context brought about a sense of guilt and blame for the affected participants.

The degree of stigma was affected by the ability to conceal symptoms of leprosy reaction which was in turn influenced by the severity and type of reaction. In both countries, people with T1R and less severe reactions who were able to conceal the leprosy reactions could gain empathy and support by calling the reactions an allergy or other non-leprosy illness. People with ENL or severe reactions who wanted to conceal their condition had to cover the signs with clothes or restrict interaction with others by staying at home. Concealment dynamics have been explored in leprosy [40,61,69–72]. A study in Indonesia found that concealment seems to increase the negative feelings and internalised stigma of affected individuals [69]. Participants in our study reported that concealment changed social interaction and had a negative psychological impact.

Leprosy reactions are a burden to affected individuals and have a significant effect on their families. Leprosy reactions caused unavoidable financial difficulty due to frequent visits to healthcare facilities, hospital admission and recurrence of leprosy reactions. The participants in both countries were financially dependent on their family members or neighbours for support. Chandler et al found that 94% of Indians with ENL lost their productivity. The study found 37.7% of households experienced catastrophic health expenditure defined as direct costs in excess of 40% of total household income and indirect costs were significantly greater than controls with leprosy but no ENL [27]. Individuals used cash savings, sold their assets, borrowed money or were gifted money to pay for treatment [27]. In this study treatment seeking and treatment of leprosy reactions contributed to the economic condition of affected individuals and their families. Absence from the workplace due to being incapacitated and frequent outpatient attendances led to a majority of Indonesians with ENL to stop work and reduce household income and the ability to afford food. Raposo et al. reported that unemployment was frequently experienced by people with Grade 2 disability and leprosy reactions [73]. Our findings suggest that financial hardship might reinforce perceptions of nutrition being linked to onset of reactions among some participants. Unemployment is significantly associated with increased odds of being undernourished or neuropathic ulceration in leprosy affected individuals [74,75]. In general, this study confirms, also for leprosy reactions and also in relation to the contexts of Indonesia and India, what advocates of the biopsychosocial model [37] and disability scholars [76–78] argue: the level of disability experienced and quality of life depends not only on the nature and severity of impairment, but is largely determined by the context in which the individual is embedded and the social, cultural and economic barriers they face.

There were several similarities, but also important differences between the two study settings with respect to stigma and financial burden. In the physical sphere, the impact on bodily functions and daily activities was comparable, but there were different coping mechanisms. The Indonesian participants preferred to stay at home, whereas the Indian participants forced themselves to remain active. In the psychological sphere, participants in both countries were sad, anxious and stressed because of their condition, but Indian participants felt more optimistic about the progress made than their Indonesian counterparts who remained very concerned. In the social sphere, the Indonesian participants gained less family support and encountered more stigma compared to Indian participants, but the latter group had more financial challenges which necessitated them to keep working. In both countries, the study participants with ENL experienced more severe consequences than those with T1R in all three spheres.

Our study had several limitations. Findings from this study might be generalizable to some extent to other hospital settings in Indonesia and India but are not necessarily representative of all individuals with experience of leprosy reactions because we did not include people who were treated at a primary health care facility. Individuals treated at the primary healthcare level might face barriers to access a referral hospital (e.g., financial barriers, lack of family support) and their experiences and the impact of the leprosy reactions on their lives might differ from those reported by individuals in this study. We may have underrepresented the views and experiences of individuals with leprosy reactions with reduced adherence to treatment. Women were also underrepresented as participants. We agree that an exploration of any differences in the experiences of men and women is important to explore and we hope to address this topic in a future manuscript.

To some extent, different characteristics of the study settings allowed us to understand more perspectives from different stakeholders, but it was difficult to compare the two study settings. In Indonesia, treatment of leprosy reactions was provided in a general hospital in an urban setting, whereas in India, treatment was provided at a specialized leprosy hospital in a rural setting. The socio-economic background of participants in both countries was different; Indian participants were poorer. Indonesians encountered more stigma despite wider access to information.

This study identified several needs. More research on identification of better treatment and new drugs to manage the reactional episodes are required. Studies on impediments to access to care and direct and indirect expenses for leprosy reaction treatment are important. A lack of understanding of leprosy reactions reported in this study shows a necessity to develop a tool to measure knowledge, attitude, and practice of the people with leprosy reactions and their caregivers. An exploration of improving methods of communication about leprosy reaction with affected individuals is also warranted. Research on the impact of leprosy reactions with larger cohorts is warranted to understand potential differences associated with gender, age, and settings. More participatory and qualitative research is needed to enhance our in-depth understanding, but in conjunction with quantitative research on the relation between reactions and mental well-being [79]. There is a need to holistically explore the role of the management of leprosy reactions and its impact on physical, psychological, and social domains of the lives of affected individuals.

The research findings provide important information for policy makers at local, national and global level about a need for early case detection of leprosy which may affect the number of people who experience leprosy reactions and sustained commitment to follow-up care for people who have completed MDT and reaction treatment.

Training on leprosy reactions for health professionals should be enhanced and implemented regularly to facilitate the early diagnosis of leprosy and leprosy reactions. Training should address the psychological and social needs of the affected individuals as well as medical aspects of the condition. Specific training topics may consist of diagnosing leprosy reactions, recognizing and dealing with risk factors or triggers, assessing an individual’s mental health, providing appropriate support, and discussing prognosis and the meaning of cure which may be different for each individual with different types of leprosy. Recognising the impact of stigma and social-economic burden should be considered when discussing treatment and monitoring response. Involving a family member when discussing the complex health needs of individuals with leprosy reaction may improve outcomes.

Strategies and interventions that could improve the lives of people affected by leprosy reactions are needed. Institutions and organisations working with leprosy affected individuals and communities should explore the potential of peer-counselling for leprosy reactions. Based on the findings that Indian participants had experienced less stigma because of having neighbours who had been affected by leprosy and some Indonesian participants use of social media and internet resources to seek information about leprosy reactions, we envision facilitating peer-support among the people with leprosy reactions within the medical and community settings through telemedicine, counselling, and rights-based interventions for affected individuals, family members, and caregivers to be worth exploring [80–83]. We recommend interventions are developed using a participatory approach and tested for their effectiveness with all relevant stakeholders.

## Conclusion

Leprosy reactions, ENL in particular, have significant impact on the physical, psychological, and social aspects of the lives of the people affected. The affected individuals experience severe physical discomfort, distress, stigma, and financial hardship. These findings provide important information about the need for early detection and sustained commitment to follow-up care for people with leprosy and leprosy reactions. More research on new drugs for reactional episodes, costings, the impact of leprosy reactions, improved training of health professionals and insights in the effectiveness of other strategies to mitigate the effects of leprosy reactions are needed.

## Supporting information

S1 Appendix Socio-demographic data. (DOCX)

S2 Appendix Interview Guide for People with Leprosy Reactions. (DOCX)
