# Supplementary material for: Understanding leprosy reactions and the impact on the lives of people affected: An exploration in two leprosy endemic countries
Source: PLoS Negl Trop Dis. 2022 Jun 13;16(6):e0010476. doi: 10.1371/journal.pntd.0010476 (PMC9191760; doi:10.1371/journal.pntd.0010476)
Supplement: S1 Appendix — (DOCX) [file pntd.0010476.s001.docx]

# S1 Appendix. Socio-demographic data

**QUESTIONNAIRE ID □□□□**

**Section 1. General information**

1. **Name:** …………………………………………
2. **Age:** …………………………………………
3. **Area of residence:** ………………………………
4. **What is your Gender?**

a. Male b. Female

1. **What is your ethnic group?**

a. Javanese b. Madurese c. Sundanese d. Other, specify………………

1. **What is your Religion?**

a. Islam b. Christian c. Catholic d. Buddhist e. Hindu f. Confucianism

1. **What is marital status?**

a. Married b. Single c. Divorced

1. **What is the highest level of education you achieved?**
2. No education b. Level of education………………………………
3. **What is your occupation?** (may circle more than one option)
4. Housewife b. Farmer c. Self-employed d. Office worker
5. Student f. Hard labor g. Seller h. Others. Specify…………………………………
6. If you are employed, **please indicate your monthly income**……………………………
7. **Who do you live with?**
8. Parents b. Spouse c. Children d. Alone f. Relatives

***Thank you very much for your response***
