# Supplementary material for: Understanding leprosy reactions and the impact on the lives of people affected: An exploration in two leprosy endemic countries
Source: PLoS Negl Trop Dis. 2022 Jun 13;16(6):e0010476. doi: 10.1371/journal.pntd.0010476 (PMC9191760; doi:10.1371/journal.pntd.0010476)
Supplement: S2 Appendix — (DOCX) [file pntd.0010476.s002.docx]

# S2 Appendix. Interview Guide for People with Leprosy Reactions

Date: _______________________________

Study site: ___________________________

Name of interviewer: __________________

Respondent ID: _______________________

Start time: __________________ End time: ________________

*This is a part of the probing interviews to explore and understand the impact of leprosy reactions on people affected in Indonesia and India, and an in-depth exploration of people experience and recommendation of management of leprosy reactions in their countries. Follow the following main topics and probe further until response saturation is elicited:*

# General questions

Leprosy is an infectious disease which can be detected on a person with skin lesions consistent with definite sensory loss or positive skin smears. This illness mainly affects the skin, the peripheral nerves, mucosa of the upper respiratory tract and the eyes. Complication of leprosy which lead to nerve function impairment, disability and neuropathic pain is called as leprosy reactions.

- 1. Can you tell me about your story on leprosy reactions?

Probe: When did it start? How did your experience start? How did it look like? What did you do? Did you talk about it with others? With whom did you talk about it?

- 1. Did you go to doctors or primary health care when feel the reactions?

Probe: When were you diagnosed having leprosy reactions by the doctor? What did they tell you about your condition? How long have you known that you had the condition? (set of questions for each experience of reactions)

- 1. What medication do you take?

Probe: Where do you get it? How much does it cost? Do you have to pay or is there insurance? Is it one medication or more? Do you use herbal medication in addition?

- 1. How do you feel about it?

# Impact of leprosy reactions

- 1. I never had leprosy reactions, how is it to have reactions?

Probe: Do they occur once or recur in episode? Why does it happen? How frequent do the reactions occur? What else do you feel when the reactions occur? Are they painful? Do they itch? How do they look?

- 1. How do you do your daily activities when the reactions occur?

Probe: Could you still go to work/school? Why/why not? Does anyone help you to do your daily activities, such as shopping, washing clothes, eating, and cooking? Who does usually help you? What do they do? Could you travel to somewhere by yourself? Why/why not?

- 1. Has any change with your behaviour or habit after living with leprosy reactions? Have you ever felt angry, stress or not comfortable with the reactions? Why/why not?
  2. How is your social life after living with the condition? How do leprosy reactions affect your daily interactions with others?

1. *How do people interact with you before and after living with leprosy? Is there any negative experience on your interaction with others after living with leprosy reactions? Can you tell me the story?*
2. *What do your family think about living with the reactions? How do they feel about it? How do they approach you? How do they behave to you after living with the reactions?*
3. What do your friends/colleagues *about living with the reactions? How do they feel about it? How do they approach you? How do they behave to you after living with the reactions?*
4. *What do your neighbour think about living with the reactions? How do they feel about it? How do they approach you? How do they behave to you after living with the reactions?*
5. What do health workers think *about living with the reactions? How do they feel about it? How do they approach you? How do they behave to you after living with the reactions?*
6. *Has any change with your relationship with your friends, family, and relatives?*
7. *What main thing you can do after living with leprosy reactions? Has any change with your habit?*
8. *When the reactions occur, do you still frequently go to mosque or church for practice your spiritual ceremony? Is it less/more visit compare to before living with the leprosy reactions?*
9. *Has any change with your employment?*
10. *Has any change with your perspective on healthcare providers?*
    1. How well have you had the opportunity to participate in the society- religious, communal, employment, facilities, education, and healthcare- after having leprosy reactions?
    2. How do leprosy reactions affect your financial condition? How can you afford life?
    3. What barriers or challenge do you encounter living with leprosy reactions? How do you manage to solve the challenges? What factors facilitate you to manage it?
    4. What are your needs and worries after **experiencing** leprosy reactions?
